# Association of Early, High Plasma-to–Red Blood Cell Transfusion Ratio With Mortality in Adults With Severe Bleeding After Trauma

**DOI:** 10.1001/jamanetworkopen.2019.12076

**Published:** 2019-09-25

**Authors:** Florian Roquet, Arthur Neuschwander, Sophie Hamada, Gersende Favé, Arnaud Follin, David Marrache, Bernard Cholley, Romain Pirracchio

**Affiliations:** 1 Service d’Anesthésie-réanimation, Hôpital Européen Georges-Pompidou, Assistance Publique–Hôpitaux de Paris, Paris, France; 2 Service de Biostatistique et Informatique Médicale, Unité INSERM UMR 1153, Université Paris Diderot, Paris, France; 3 Service d’Anesthésie-réanimation, Centre Hospitalier Universitaire Bicêtre, Université Paris Sud, Assistance Publique–Hôpitaux de Paris, Le Kremlin Bicêtre, France

## Abstract

**Importance:**

Optimal transfusion management is crucial when treating patients with trauma. However, the association of an early, high transfusion ratio of fresh frozen plasma (FFP) to packed red blood cells (PRBC) with survival remains uncertain.

**Objective:**

To study the association of an early, high FFP-to-PRBC ratio with all-cause 30-day mortality in patients with severe bleeding after trauma.

**Design, Setting, and Participants:**

This cohort study analyzes the data included in a multicenter national French trauma registry, Traumabase, from January 2012 to July 2017. Traumabase is a prospective, active, multicenter adult trauma registry that includes all consecutive patients with trauma treated at 15 trauma centers in France. Overall, 897 patients with severe bleeding after trauma were identified using the following criteria: (1) received 4 or more units of PRBC during the first 6 hours or (2) died from hemorrhagic shock before receiving 4 units of PRBC.

**Exposures:**

Eligible patients were divided into a high-ratio group, defined as an FFP-to-PRBC ratio more than 1:1.5, and a low-ratio group, defined as an FFP-to-PRBC ratio of 1:1.5 or less. The ratio was calculated using the cumulative units of FFP and PRBC received during the first 6 hours of management.

**Main Outcomes and Measures:**

A Cox regression model was used to analyze 30-day survival with the transfusion ratio as a time-dependent variable to account for survivorship bias.

**Results:**

Of the 12 217 patients included in the registry, 897 (7.3%) were analyzed (median [interquartile range] age, 38 (29-54) years; 639 [71.2%] men). The median (interquartile range) injury severity score was 34 (22-48), and the overall 30-day mortality rate was 33.6% (301 patients). A total of 506 patients (56.4%) underwent transfusion with a high ratio and 391 (43.6%) with a low ratio. A high transfusion ratio was associated with a significant reduction in 30-day mortality (hazard ratio, 0.74; 95% CI, 0.58-0.94; *P* = .01). When only analyzing patients who had complete data, a high transfusion ratio continued to be associated with a reduction in 30-day mortality (hazard ratio, 0.57; 95% CI, 0.33-0.97; *P* = .04).

**Conclusions and Relevance:**

In this analysis of the Traumabase registry, an early FFP-to-PRBC ratio of more than 1:1.5 was associated with increased 30-day survival among patients with severe bleeding after trauma. This result supports the use of early, high FFP-to-PRBC transfusion ratios in patients with severe trauma.

## Key Points

**Question:**

Is an early, high transfusion ratio of fresh frozen plasma to packed red blood cells associated with 30-day mortality in patients with severe bleeding after trauma?

**Findings:**

In this cohort study of 897 patients in the French national trauma registry Traumabase, an early, high transfusion ratio of fresh frozen plasma to packed red blood cells was associated with increased 30-day mortality in patients with severe bleeding after trauma.

**Meaning:**

These results support an early, high transfusion ratio of fresh frozen plasma to packed red blood cells as part of the hemostatic resuscitation strategy in severe trauma.

## Teaser

This cohort study analyzes data from the French national trauma registry Traumabase to examine whether an early, high ratio of fresh frozen plasma to packed red blood cells is associated with all-cause 30-day mortality in adults with severe bleeding after trauma.

## Introduction

Deaths secondary to trauma accounted for 9% of global mortality in 2012, ie, approximately 5 million deaths that year.^1^ Although mortality among patients who experienced trauma is typically associated with central nervous system injuries, bleeding is the second leading cause of death^2^ and is considered the predominant cause of preventable death after hospital admission.^3,4^ Indeed, mortality attributable to hemorrhage accounts for 30% to 40% of overall mortality after major trauma,^5^ of which 7% is considered preventable.^6,7^ Acute bleeding may cause early death through exsanguination or secondary hypoperfusion and coagulopathy, which can precipitate multiple organ dysfunction and later mortality.^8,9,10^ Therefore, the management of acute hemorrhage is based on the following: (1) controlling the source of bleeding, (2) restoring adequate organ perfusion, and (3) preventing or treating coagulation disorders. Hence, in addition to hemodynamic resuscitation, patients with severe bleeding after trauma often require the transfusion of packed red blood cells (PRBC) to restore oxygen transport and tissue oxygenation and of fresh frozen plasma (FFP) and platelets to prevent or correct coagulation disorders, such as trauma-induced coagulopathy.^11^

Proportional blood component transfusion is intended to approach whole blood transfusion.^12^ Since the advent of apheresis, whole blood transfusion has been abandoned in routine civilian clinical practice but not in military medicine.^13^ In 2005, the US Department of Defense initiated the codification of damage control resuscitation and included a 1:1 FFP-to-PRBC ratio as a fundamental principle for clinical practice.^14^ Based on data from the Joint Theater Trauma Registry,^15^ military medicine was the first to report the benefit of a higher FFP-to-PRBC ratio in patients with a penetrating trauma. Consequently, it has been suggested that early administration of FFP and PRBC in a comparable amount may prevent the development of coagulopathy and thereby improve early survival.^16^ In 2008, Duchesne et al^17^ published the first retrospective observational civilian study on this topic. Subsequently, several retrospective civilian and military studies have been published, culminating in the completion of a large multicenter randomized trial in 2015 (the Pragmatic, Randomized Optimal Platelet and Plasma Ratios Trial).^18^ However, this trial did not show any difference between the 2 transfusion ratios on the primary end point.^18^ Yet, in 2016, the National Clinical Guideline Centre of the United Kingdom recommended the ratio of 1 unit of plasma to 1 unit of red blood cells to replace fluid volume in adult trauma care.^19^ Although these recommendations are now widely applied in both civilian and military trauma centers,^20,21^ there is still limited evidence supporting the superiority of high (ie, close to 1) vs lower FFP-to-PRBC ratios. Indeed, most studies are observational, some of them comparing the outcome before and after the implementation of a massive transfusion protocol.^22,23,24,25,26^ These studies were potentially biased if they compare FFP-to-PRBC ratios between survivors and nonsurvivors without accounting for confounding factors.^16,27,28^ In addition, these studies were frequently underpowered, and most of them systematically discarded patients receiving fewer than 10 units of PRBC during the first 24 hours. Finally, many studies define high and low FFP-to-PRBC ratios by averaging the total number of units of FFP and PRBC transfused during the first 24 hours.^13,23,29,30,31,32^ Hence, these studies pooled patients for whom the strategy was always to transfuse FFP and PRBC in a similar amount with patients for whom units of FFP were transfused later because of persistent bleeding or confirmed coagulation disorders. Thus, despite several studies, the optimal FFP-to-PRBC ratio remains uncertain.

The goal of the present study was to use a large French multicenter prospective trauma registry to evaluate the association of an early (ie, first 6 hours), high FFP-to-PRBC transfusion ratio (ie, a mean of >2 FFP to 3 PRBC^33^) with 30-day survival in patients with severe bleeding after trauma while accounting for confounders.

## Methods

The conduct of this retrospective cohort study and access to the data was approved by the Scientific Committee of the Traumabase. In this noninterventional registry with patients included in an emergency setting, a waiver of informed consent was provided. The Strengthening the Reporting of Observational Studies in Epidemiology (STROBE) reporting guideline was used to ensure the reporting of this registry cohort study.^34^

### Patients

We retrospectively analyzed the data from the Traumabase registry collected between January 2012 and July 2017. All consecutive patients with severe bleeding occurring within the first 6 hours of hospital admission after trauma were included in the analysis. In this registry, severe bleeding is defined as the need for an early transfusion of at least 4 units of PRBC within the first 6 hours after hospital admission.^35,36^ Patients who died of hemorrhagic causes before receiving 4 units of PRBCs were also included. The patients who died on the scene or during hospital transfer without any blood transfusion were not included.

### Traumabase

The Traumabase registry was launched in January 2012.^37^ Traumabase is an active, multicenter, prospective registry of all adult patients admitted to 1 of 15 participating French level I^38^ trauma centers (13 public civilian teaching hospitals and 2 military teaching hospitals)^39,40,41^ at the time of the study.

### Data Collection

Traumabase includes data on the prehospital phase, trauma bay and initial surgery, and intensive care unit (ICU) stay as well as status at hospital discharge. The data are prospectively collected by medical staff and research technicians at their respective hospital sites. Data collection includes information on demographic characteristics; injury pattern; medication before trauma; prehospital and in-hospital management, including initial surgery, relevant biological results, and transfusion; and patient outcome. This registry is in accordance with all requirements from the advisory committee for the processing of research information in the field of health, the French National Commission on Computing and Liberty. It also meets the requirements of the local and national ethics committee.

### Transfusion Ratios

As described in the Prospective, Multicenter, Major Trauma Transfusion Study,^42^ an individual transfusion ratio trajectory varies with time. We used transfusion ratio as a time-varying variable, and it was computed for each period of interest. In Traumabase, the cumulative number of blood components transfused is available at different points. For this study, transfusion ratios were calculated at 2 different points: first, blood products transfused during initial resuscitation in the trauma bay before transfer to computed tomographic imaging or the operating room (ie, during the first hour) and, second, the total number of blood products received by hour 6 (ie, the blood products received during the first 6 hours, including those received during the first hour). Consequently, each patient surviving the first 6 hours had 2 data points. The cumulative units of FFP and PRBC for each period until hour 6 were used to calculate an average transfusion ratio. In our study, a high ratio was defined as an FFP-to-PRBC ratio of more than 1:1.5; a low ratio was defined as an FFP-to-PRBC ratio of 1:1.5 or less. This cutoff was proposed by several authors^43,44^ and a 2018 meta-analysis.^33^

### Outcome Measures

The primary outcome measure was 30-day survival. Secondary outcome measures included 24-hour survival, lengths of ICU and hospital stays, total amount of PRBC transfused during the first 24 hours, and the length of mechanical ventilation.

### Statistical Analysis

Data are summarized as median (interquartile range [IQR]) for continuous variables and count (percentage) for categorical variables. The Fisher exact test was used to compare categorical data between ratio groups and the Wilcoxon rank sum test to compare continuous variables. Missing data are presented in eTable 1 in the Supplement.

For each ratio, the actuarial survival was described using the Kaplan-Meier estimator and compared using the log-rank test. To account for the risk of survival bias, a multivariable Cox proportional hazard regression model, with the transfusion ratio treated as a time-dependent variable, was used to identify independent factors associated with 30-day and 24-hour mortalities.^45^ Hazard ratios (HRs) were provided with 95% CIs. The statistical power was calculated a posteriori using the R package powerSurvEpi (R Project for Statistical Computing). Model goodness-of-fit was evaluated using the area under the receiver operating characteristics curve (risksetROC package).^46^ A multivariate linear regression model was used to identify all independent factors associated with the amount of PRBC transfused during the first 24 hours, the length of mechanical ventilation in survivors, and the lengths of ICU and hospital stays in survivors.

The variables included in the models were selected from those available in the database and chosen for their clinical or biological relevance. More details on the adjustment variables are provided in the eMethods in the Supplement. The primary analysis was performed after multiple imputation of missing data (MICE package for R).^47^ Center effect was accounted for by adding the center as a random effect in the multilevel Cox proportional hazard models. A procedure based on the minimization of the Akaike information criterion was used for model optimization. All clinically relevant interaction terms were tested and included in the model if statistically significant (ie, *P* < .05).

Prespecified sensitivity analyses included analysis on complete cases, 6-hour survival analysis, and analyses with FFP-to-PRBC ratio thresholds ranging from 1:2 to 4:5. All analyses were performed with R software version 3.3.3 for Windows, with the statistical significance level set at *P* < .05. All tests were 2-tailed.

## Results

### Patients

**Figure 1.**
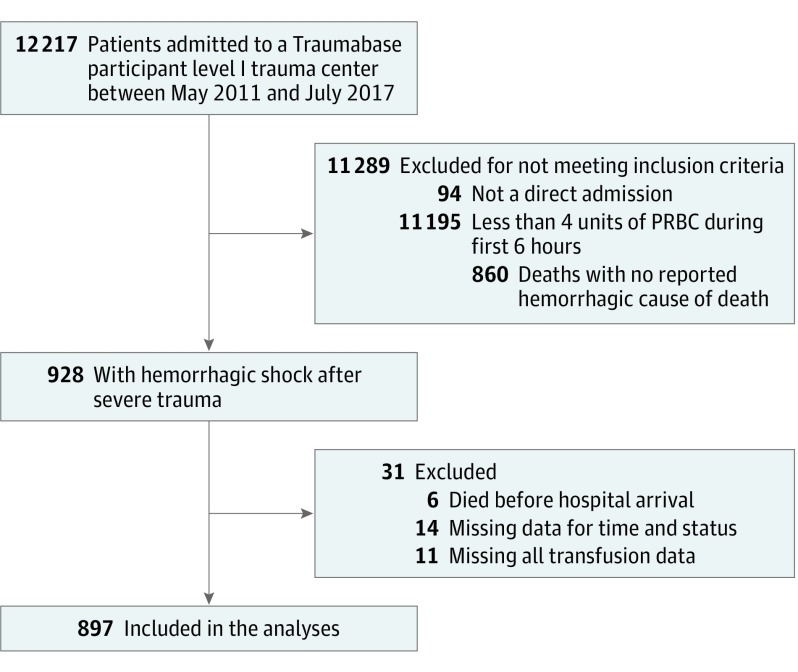
Study Flowchart PRBC indicates packed red blood cells.

Between January 2012 and July 2017, 12 217 patients were entered in the Traumabase registry. Based on our inclusion and exclusion criteria, a total of 897 patients (7.3%) were included in the analysis: 639 (71.2%) were male, with a median age of 38 years (IQR, 29-54 years; range, 13-99 years) (Figure 1). Patient characteristics are presented in Table 1, stratified by ratio group. Overall patient characteristics are presented in eTable 2 in the Supplement. The median (IQR) injury severity score was 34 (22-48), and the overall 30-day mortality rate was 33.6% (301 patients). A total of 131 patients (14.6%) experienced penetrating trauma. Urgent surgery (ie, surgery during the first 24 hours) was needed for 696 patients (77.6%). The median (IQR) number of units of PRBC transfused during the first 6 hours was 6 (4-9). A total of 506 patients (56.4%) underwent transfusion with a high ratio and 391 (43.6%) with a low ratio. The number of patients receiving a high and a low transfusion ratio during each period is reported in Table 2. The 6-hour mortality rate was 12.7% (114 of 897), with 72 patients (18.4%) in the low-ratio group and 42 patients (8.3%) in the high-ratio group. Of the 72 patients in the low-ratio group who died, 22 (31.5%) did not receive any FFP, and 66 (96.7%) received at least 4 units of PRBC.

**Table 1.** Patient Characteristics According to the Transfusion Ratio Received at the Sixth Hour^a^

Characteristic	No. (%)		*P* Value
	Low-Ratio Group (n = 391)	High-Ratio Group (n = 506)	
Died	146 (37.3)	155 (30.6)	.04
Age, median (IQR), y	39 (26-54)	36 (25-54)	.10
Men	288 (73.7)	351 (69.4)	.18
Treatment history			
Anticoagulants	13 (3.3)	11 (2.2)	.30
Antiplatelet drugs	18 (4.6)	22 (4.3)	.87
SAPS II, median (IQR)	53 (37-73)	51 (37-67)	.20
ISS, median (IQR)	34 (21-50)	34 (22-45)	.62
GCS <9	132 (33.8)	190 (37.5)	.26
Penetrating trauma	58 (14.8)	73 (14.4)	.92
Intentionality			
Unintentional trauma	216 (55.2)	293 (57.9)	.56
Trauma after assault	38 (9.7)	56 (11.1)	
Self-inflicted trauma	104 (26.6)	122 (24.1)	
Prothrombin time, median (IQR), %	51 (34-65)	47 (34-61)	.19
Blood lactate concentration, median (IQR), mg/dL	45.0 (25.2-82.0)	40.5 (25.2-68.5)	.24
Operative intervention within 24 h			
Orthopedic surgery	153 (39.1)	216 (42.7)	.31
Vascular surgery, radio interventional	105 (26.9)	150 (29.6)	.37
Neurosurgery	19 (4.9)	43 (8.5)	.03
Abdominal surgery	83 (21.2)	117 (23.1)	.52
Thoracic surgery	34 (8.7)	28 (5.5)	.08
Prehospital or ED			
Vasopressor	179 (45.8)	260 (51.4)	.11
Cardiac arrest	104 (26.8)	99 (19.6)	.02
Prehospital highest HR, median (IQR), beats/min	105 (81-125)	108 (84-124)	.59
Fibrinogen concentrate, median (IQR), g	3 (0-3)	3 (1.5-4.5)	<.001
Platelets concentrate, median (IQR), units	0 (0-1)	1 (0-2)	<.001
Tranexamic acid administration	261 (66.8)	382 (75.5)	.008
Capillary hemoglobin, median (IQR), g/dL	9.2 (7.8-11.1)	9.5 (8.0-11.0)	.63
Prehospital lowest SBP, median (IQR), mm Hg	91 (67-113)	95 (70-116)	.50
Prehospital tracheal intubation	226 (57.8)	343 (67.8)	.002
Lowest core temperature, median (IQR), ° C	35.0 (33.6-35.8)	34.5 (33.5-35.4)	.004
Fluid replacement during first 6 h, median (IQR), mL	1500 (988-2000)	1250 (1000-2000)	.77

Abbreviations: ED, emergency department; GCS, Glasgow Coma Scale; HR, heart rate; IQR, interquartile range; ISS, injury severity score; SAPS II, Simplified Acute Physiology Score; SBP, systolic blood pressure.

SI conversion factors: To convert blood lactate concentration to millimoles per liter, multiply by 0.111; capillary hemoglobin to grams per liter, multiply by 10.0.

^a^ High ratio indicates a fresh frozen plasma to packed red blood cells ratio of more than 1:1.5; low ratio, 1:1.5 or less.

**Table 2.** Distribution of 897 Patients by Average Ratio Received During the Considered Periods^a^

Distribution and Outcome	No./Total No. (%)	
	Low-Ratio Group	High-Ratio Group
**Average Ratio During the First Hour**		
Patients exposed	521/897 (58.1)	376/897 (41.9)
30-d death	191/521 (36.6)	110/376 (29.3)
**Average Ratio During the First 6 Hours**		
Patients exposed	391/897 (43.6)	506/897 (56.4)
30-d death	146/391 (37.3)	155/506 (30.6)

^a^ High ratio indicates a fresh frozen plasma to packed red blood cells ratio of more than 1:1.5; low ratio, 1:1.5 or less.

### Primary Outcome

**Figure 2.**
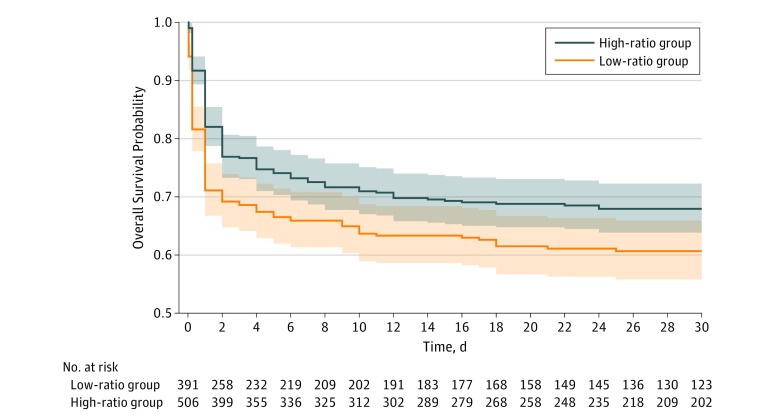
Kaplan-Meier Survival Curves for the Probability of Survival During the First 30 Days Following Hospital Admission According to the Transfusion Ratio Used During the First 6 Hours High ratio indicates a fresh frozen plasma to packed red blood cells ratio of more than 1:1.5; low ratio, 1:1.5 or less. Shaded areas around curves represent 95% CIs. Log-rank test for 30-day survival, *P* = .006.

As illustrated by the Kaplan-Meier curves, a high transfusion ratio was associated with increased 30-day survival (log-rank *P* = .006) (Figure 2). After adjusting for potential confounders, a high FFP-to-PRBC ratio was still associated with an increase in 30-day survival (HR, 0.74; 95% CI, 0.58-0.94; *P* = .01) (Table 3). All relevant interactions are presented in eTable 3 in the Supplement. The goodness-of-fit of the model as evaluated by the area under the receiver operating characteristics curve was 0.86 (eFigure 1 in the Supplement). The statistical power computed a posteriori was 78%. When only analyzing the 594 complete cases, the reduction in the risk of death during the first 30 days with a high FFP-to-PRBC ratio also reached statistical significance (HR, 0.57; 95% CI, 0.33-0.97; *P* = .04) (Table 3). The association of a high transfusion ratio and mortality at 6 hours was similar but failed to be statistically significant (complete cases: HR, 0.71; 95% CI, 0.35-1.43; *P* = .30; after multiple imputation: HR, 0.91; 95% CI, 0.61-1.35; *P* = .60). Results are reported in eTable 4 in the Supplement. The results of the sensitivity analyses with different FFP-to-PRBC ratios are presented in eFigure 2 in the Supplement.

**Table 3.** Adjusted Cox Model for 30-day Survival

Factor^a^	Complete Cases (n = 594)		After Multiple Imputation (n = 897)	
	HR (95% CI)	*P* Value	HR (95% CI)	*P* Value
SAPS II	1.01 (0.98-1.03)	.44	1.04 (1.03-1.04)	<.001
ISS	1.01 (1.00-1.02)	.12	1.01 (0.99-1.01)	.10
Intentionality				
Self-inflicted vs unintentional	0.58 (0.40-0.85)	.005	0.67 (0.49-0.91)	.01
Assault vs unintentional	0.22 (0.11-0.44)	<.001	0.39 (0.24-0.65)	<.001
GCS	0.76 (0.65-0.88)	<.001	0.93 (0.89-0.97)	<.001
Prehospital or ED cardiac arrest	2.35 (1.61-3.43)	<.001	2.39 (1.77-3.24)	<.001
Prothrombin time	1.34 (1.09-1.65)	.006	0.97 (0.97-0.98)	<.001
Capillary hemoglobin, g/dL	0.89 (0.83-0.97)	.005	0.94 (0.89-0.99)	.04
Temperature	1.24 (0.99-1.55)	.07	0.83 (0.75-0.92)	<.001
Fibrinogen concentrate administration	0.72 (0.62-0.82)	<.001	0.93 (0.89-0.98)	.003
Prehospital or ED vasopressor	0.63 (0.43-0.92)	.02	0.98 (0.73-1.31)	.88
Fluid replacement first 6 h (crystalloids and colloids), 1-mL increase	0.99 (0.99-0.99)	.005	0.99 (0.99-0.99)	.01
Platelets concentrate, 1-unit increase	1.58 (1.21-2.07)	<.001	1.06 (0.96-1.17)	.22
Orthopedic surgery	0.32 (0.20-0.50)	<.001	0.35 (0.24-0.50)	<.001
Vascular surgery	0.57 (0.40-0.82)	.002	0.66 (0.51-0.86)	.002
High ratio vs low ratio^b^	0.57 (0.33-0.97)	.04	0.74 (0.58-0.94)	.01

Abbreviation: ED, emergency department; GCS, Glasgow Coma Scale; HR, hazard ratio; ISS, injury severity score; SAPS II, Simplified Acute Physiology Score.

^a^ Relevant interactions appear in eTable 3 in the Supplement.

^b^ High ratio indicates a fresh frozen plasma to packed red blood cells ratio of more than 1:1.5; low ratio, 1:1.5 or less.

### Secondary Outcomes

Overall, the 24-hour mortality rate was 22.7% (204 patients): 91 deaths (18.0%) in the high-ratio group and 113 deaths (28.9%) in the low-ratio group (HR, 0.79; 95% CI, 0.58-1.06; *P* = .11) (eTable 5 in the Supplement). In multivariate analysis, the total units of PRBC transfused during the first 24 hours was not associated with the transfusion ratio: the high-ratio group received a median (IQR) of 6 (4-8.5) units of PRBC, and the low ratio group received a median (IQR) of 6 (4-7) units of PRBC (odds ratio [OR], 0.99; 95% CI, 0.93-1.06; *P* = .81). This was also true in the population of patients who survived the first 24 hours (median [IQR], 6 [4-9] units of PRBC in the high-ratio group vs 6 [4-8] units of PRBC in the low-ratio group; OR, 0.98; 95% CI, 0.92-1.05; *P* = .52). In multivariate analysis, the total units of FFP transfused during the first 24 hours was associated with the transfusion ratio (median [IQR], 6 [4-9] units of FFP in the high-ratio group vs 3 [2-4] units of FFP in the low ratio group; OR, 23.86; 95% CI, 14.22-40.33; *P* < .001). Among survivors, the total units of FFP transfused during the first 24 hours was also associated with the transfusion ratio (median [IQR], 6 [4-9] units of FFP in the high-ratio group vs 3 [2-4] units of FFP in the low-ratio group; OR, 21.26; 95% CI, 13.51-33.47; *P* < .001). In 584 patients discharged from the hospital, the FFP-to-PRBC ratio was not associated with the length of ICU stay (median [IQR], 16 [8-32] days in the high-ratio group vs 11 [4-24] days in the low-ratio group; OR, 1.25; 95% CI, 0.28-5.40; *P* = .31), the length of hospital stay (median [IQR], 34 [19-60] days in the high-ratio group vs 28 [13-53] days in the low-ratio group; OR, 2.60; 95% CI, 0.01-939.90; *P* = .74), or the duration of mechanical ventilation (median [IQR], 5 [2-13] days in the high-ratio group vs 3.5 [1-12] days in the low-ratio group; OR, 1.02; 95% CI, 0.17-6.27; *P* = .64).

## Discussion

While massive bleeding remains a leading cause of preventable death after trauma, there is still limited scientific evidence on how to optimally transfuse these patients.^48^ The only large-scale multicenter randomized clinical trial (the Pragmatic, Randomized Optimal Platelet and Plasma Ratios Trial)^18^ on this topic, to our knowledge, was conducted in the United States and compared 2 platelet-to-FFP-to-PRBC transfusion ratios (1:1:1 vs 1:1:2). This trial did not show any difference in mortality at 24 hours and 30 days among 701 patients with severe trauma, although deaths by exsanguination occurring in the first 24 hours were less frequent in the group receiving a 1:1:1 transfusion ratio. Based on a large multicenter observational trauma registry, we were able to show that a high transfusion ratio (FFP-to-PRBC ratio >1:1.5) was associated with increased 30-day survival. Consistently, previous studies tended to show a benefit of early transfusion of units of FFP.^16,49^ This hypothesis is supported by a 2018 superiority phase 3 randomized controlled trial (Prehospital Air Medical Plasma Trial).^50^ In this trial, a prehospital FFP transfusion in patients at risk of hemorrhagic shock after trauma resulted in lower 30-day mortality compared with the standard of care, with a number needed to treat of 10 patients.^50,51^ However, this result should be mitigated because a 2018 randomized clinical trial^52^ did not find any benefit associated with an early prehospital FFP transfusion in an urban environment.

The estimated association of the transfusion ratio with mortality depends on the population under study as well as on the cutoff used to define high and low ratios. A transfusion of at least 4 units of PRBC during the first 6 hours of hospital admission was required to enter our study, while many other studies used massive transfusion as inclusion criteria. Massive transfusion is sometimes defined as 10 units of PRBC transfused during the first 24 hours or 1 unit of PRBC per hour for 4 consecutive hours.^20^ While the latter definition is close to our definition, we believe that the former may be flawed. First, this definition aggregates heterogeneous patient groups and transfusion strategies. Patients who experienced trauma may bleed immediately because of the injury but may also bleed later because of surgery or coagulation disorders. The transfusion strategy is also likely evolving during the first 24 hours. Such a dynamic transfusion pattern is not accounted for when considering large time windows, such as the first 24 hours.^53,54^ Second, this definition increases the risk of survival bias because the patients with most severe trauma may die before receiving 10 units of blood.^21,55^ Third, other transfusion parameters (ie, transfusion requirements in the first 6 hours or transfusion of at least 3 units of PRBC in 1 hour or 5 units of PRBC in 4 hours) have a stronger association with mortality.^54,55^ While an FFP-to-PRBC ratio cutoff of 1:2 was used in several studies^17,56,57,58^ to define low and high transfusion ratios, we opted for a threshold of 1:1.5. This value was proposed by several authors,^43,44^ including a 2018 meta-analysis,^33^ and as suggested in our sensitivity analyses (eFigure 2 in the Supplement), it may be associated with an improvement in 30-day mortality.

Trauma-related mortality also depends on trauma mechanism. In their clinical presentation, management, and outcomes, blunt trauma and penetrating trauma present substantial differences.^59^ In contrast to military studies or to civilian studies performed in the United States,^25,44,49,57^ penetrating trauma only accounted for 15% of the injury mechanisms in our population. In our series, the proportion of penetrating trauma is close to that observed in the German trauma registry (10%).^60^ Our results are in contrast with those of Rowell et al,^61^ who reported a lack of benefit on 30-day survival of a high FFP-to-PRBC ratio in blunt trauma. However, in the study by Rowell et al,^61^ the definition of a high FFP-to-PRBC transfusion ratio was 1:2. In contrast to Rowell et al^61^ but consistent with our results, a Swiss observational study^62^ reported a survival benefit at 12 and 24 hours after a blunt trauma when using a high FFP-to-PRBC ratio defined with a threshold of 1:1.5.^62^ Unlike previous studies,^63,64^ we decided not to exclude patients with a serious traumatic brain injury (TBI) or with an isolated TBI for 2 reasons. First, Peiniger et al^60^ did not show any difference in survival between patients with or without TBI according to the transfusion ratio strategy. Second, TBI is the leading cause of death after trauma.^65^ In addition, TBIs are associated with significant hemostatic disorders in approximately 17% to 30% of patients.^66,67,68^ Thus, patients with TBI are particularly prone to severe hemorrhage. In this context, severe bleeding may cause death by exsanguination or contribute to aggravating brain lesions.^66^ Hence, patients with TBI may particularly benefit from transfusion ratios close to 1, although we were not able to specifically target this subpopulation in our analysis.

### Limitations

Our study has some limitations. First, studies in severe trauma are prone to survivorship bias.^58,69^ In our series, the mortality rate during the first 6 hours was 12.7% (114 of 897), with 18.4% (72 of 391) in the low-ratio group at the sixth hour (of whom 22 did not receive any FFP and 66 received ≥4 units of PRBC) and 8.3% (42 of 506) in the high-ratio group. To reduce survivorship bias, we performed a Cox regression with ratio as a time-dependent covariate, and we found a difference in survival between the 2 groups. Second, although data quality was regularly enforced by data managers, missing data were still present. They were handled using multivariate imputation by chained equations.^70^ This method has been shown to be statistically valid and robust to model misspecification when data are missing at random or completely at random. In addition, our main results were confirmed with an analysis of complete cases.

## Conclusions

In this study, a transfusion strategy based on an early FFP-to-PRBC ratio of more than 1:1.5 was associated with decreased 30-day mortality among patients with severe bleeding after trauma. Further studies are needed to identify optimal, personalized, and dynamic transfusion strategies to help clinicians adjust the transfusion strategy in real time.

## Notes

Supplement. **eMethods.** Variable Selection for Cox Regression Model **eTable 1.** Table of Missing Data According to the Transfusion Ratio at the Sixth Hour **eTable 2. **Overall Patient Characteristics **eTable 3.** Adjusted Cox Model for 30-d Survival Relevant Interactions **eTable 4.** Adjusted Cox Model for 6-h Survival **eTable 5.** Adjusted Cox Model for 24-h Survival **eFigure 1.** Receiver Operating Characteristics (ROC) Curve of the Model Selected for 30-d Survival **eFigure 2.** Sensitivity Analyses With Different Ratio Cutoffs (Cox Model on Complete Cases) **eReferences.**
