# Errors in Key Points and Corresponding Author Address

**DOI:** 10.1001/jamanetworkopen.2019.15468

**Published:** 2019-10-18

The Original Investigation titled “Association of Early, High Plasma-to–Red Blood Cell Transfusion Ratio With Mortality in Adults with Severe Bleeding After Trauma,”^1^ published September 25, 2019, included an error in the Key Points section. Findings should have read, “In this cohort study of 897 patients in the French national trauma registry Traumabase, an early, high transfusion ratio of fresh frozen plasma to packed red blood cells was associated with increased 30-day survival in patients with severe bleeding after trauma.” The corresponding author’s email address should have been romainpirracchio@yahoo.fr. The following 4 names should have been included in the Traumabase Group Members: Delphine Garrigue, Jean-Luc Hanouz, Eric Meaudre, and Julien Pottecher. This article has been corrected.^1^
