# Supplementary material for: Association of Early, High Plasma-to–Red Blood Cell Transfusion Ratio With Mortality in Adults With Severe Bleeding After Trauma
Source: JAMA Netw Open. 2019 Sep 25;2(9):e1912076. doi: 10.1001/jamanetworkopen.2019.12076 (PMC6763975; doi:10.1001/jamanetworkopen.2019.12076)
Supplement: Supplement. — eMethods. Variable Selection for Cox Regression Model eTable 1. Table of Missing Data According to the Transfusion Ratio at the Sixth Hour eTable 2. Overall Patient Characteristics eTable 3. Adjusted Cox Model for 30-d Survival Relevant Interactions eTable 4. Adjusted Cox Model for 6-h Survival eTable 5. Adjusted Cox Model for 24-h Survival eFigure 1. Receiver Operating Characteristics (ROC) Curve of the Model Selected for 30-d Survival eFigure 2. Sensitivity Analyses With Different Ratio Cutoffs (Cox Model on Complete Cases) eReferences. [file jamanetwopen-2-e1912076-s001.pdf]

Supplementary Online Content

Roquet F, Neuschwander A, Hamada S, et al; Traumabase Group. Association of early, high plasma-to–red blood cell transfusion ratio with mortality in adults with severe bleeding after trauma. *JAMA Netw Open*. 2019;2(9):e1912076. doi:10.1001/jamanetworkopen.2019.12076

eMethods. Variable Selection for Cox Regression Model

eTable 1. Table of Missing Data According to the Transfusion Ratio at the Sixth Hour

eTable 2. Overall Patient Characteristics

eTable 3. Adjusted Cox Model for 30-d Survival Relevant Interactions

eTable 4. Adjusted Cox Model for 6-h Survival

eTable 5. Adjusted Cox Model for 24-h Survival

eFigure 1. Receiver Operating Characteristics (ROC) Curve of the Model Selected for 30-d Survival

eFigure 2. Sensitivity Analyses With Different Ratio Cutoffs (Cox Model on Complete Cases)

eReferences.

This supplementary material has been provided by the authors to give readers additional information about their work.

© 2019 Roquet F et al. *JAMA Network Open.*

eMethods. Variable Selection for Cox Regression Model

The variables included in the models were selected from those available in the database and chosen for their clinical or biological relevance. We included the context of the trauma and its characteristics. Risk factors and biological markers of coagulopathy were added, and we aimed to include all clinical or biological parameters that were identified as independent predictors of mortality in previously published studies. We performed a correlation analysis for quantitative covariates and kept clinically relevant interactions (e.g. the interaction between SAPSII and GCS). Based on this selection procedure, the variables included in the models were : age, gender, anticoagulant or antiplatelet medication history, trauma characteristics including intentionality and mechanism, Injury Severity Score (ISS)[1], Simplified Acute Physiology Score II (SAPS II)[2], initial Glasgow Coma Scale (GCS)[3], lowest body temperature, prehospital tracheal intubation, prehospital cardiac arrest or cardiac arrest at hospital admission, prehospital lactatemia, prehospital capillary hemoglobin measurement, prehospital clinical parameters including lowest systolic arterial blood pressure and highest heart rate, prothrombin time (PT) at hospital admission, surgery (orthopedic surgery, vascular surgery, neurosurgery, thoracic surgery, abdominal surgery) and interventional radiology, fluid replacement of the first 6 hours (both colloids and crystalloids), need for vasopressor, tranexamic acid administration, platelet, and fibrinogen concentrate transfusion.

© 2019 Roquet F et al. *JAMA Network Open.*

eTable 1. Table of Missing Data According to the Transfusion Ratio at the Sixth Hour

No. (%) of data missing	Low ratio	High ratio	Overall
	(n=391)	(n=506)	(n=897)
Death missing	0	0	0
Age - years	0	0	0
Gender male	0	0	0
Treatment history	38 (9.9%)	39(7.7%)	77 (8.6%)
SAPS II	0	0	0
ISS	7 (1.8%)	13 (2.6%)	20 (2.2%)
GCS <9	0	0	0
Mechanism penetrating	0	0	0
Intentionality	33 (8.4%)	35 (6.9%)	68 (7.6%)
Prothrombin time (%)	23 (5.9%)	12 (2.4%)	35 (3.9%)
Lactatemia - mmol/L	35 (8.9%)	21 (4.2%)	56 (6.2%)
Early operative intervention <24h	0	0	0
Prehospital or emergency room vasopressor	0	0	0
Prehospital or emergency room cardiac arrest	0	0	0
Prehospital highest heart rate - per minute	18 (4.6%)	36 (7.1%)	54 (6.0%)
Fibrinogen concentrate - g	0	0	0
Platelets concentrate	0	0	0
Tranexamic acid administration	6 (1.5%)	3 (0.6%)	9 (1.0%)
Capillary hemoglobin - g/dL	4 (1.0)	11 (2.2%)	15 (1.7%)
Prehospital lowest SBP - mmHg	7 (1.7%)	11 (2.2%)	18 (2.0%)
Prehospital tracheal intubation	0	1 (0.2%)	1 (0.1%)
Lowest core temperature - Celsius	29 (7.4%)	36 (7.1%)	65 (7.2%)
Fluid replacement first 6 hours- mL	27 (6.9%)	50 (9.9%)	77 (8.6%)

© 2019 Roquet F et al. *JAMA Network Open.*

eTable 2. Overall Patients Characteristics

	**Overall population**
	n=897
No. (%) of death	301 (33.6%)
Age, median [IQR], years	38 [26-54]
Gender male, No. (%)	639 (71.2%)
Treatment history, No. (%)	
Anticoagulants	24 (2.7%)
Antiplatelet drugs	40 (4.5%)
SAPS II, median [IQR]	51 [37-69]
ISS, median [IQR]	34 [22-48]
GCS <9, No. (%)	322 (35.9%)
No. (%) of penetrating trauma	131 (14.6%)
Intentionality, No. (%)	
Unintentional trauma	509 (56.7%)
Trauma after assault	94 (10.5%)
Self-inflicted trauma	226 (25.2%)
Prothrombin time, median [IQR], %	49 [34-63]
Lactatemia, median [IQR], mmol/L	4.7 [2.8-8.3]
Early operative intervention <24h, No. (%)	
Orthopedic surgery	369 (41.1%)
Vascular surgery - radio interventional	255 (28.4%)
Neurosurgery	62 (6.9%)
Abdominal surgery	200 (22.3%)
Thoracic surgery	62 (6.9%)
Prehospital or ER vasopressor, No. (%)	439 (48.9%)
Prehospital or ER cardiac arrest, No. (%)	203 (22.6%)
Prehospital highest HR, median [IQR], per minute	106 [83-125]
Fibrinogen concentrate, median [IQR], g	3 [0-4.5]
Platelets concentrate, median [IQR]	1 [0-2]
Tranexamic acid administration, No. (%)	643 (71.7%)
Capillary hemoglobin, median [IQR], g/dL	9.5 [8.0-11.0]
Prehospital lowest SBP, median [IQR], mm Hg	92 [70-115]
Prehospital tracheal intubation, No. (%)	569 (63.4%)
Lowest core temperature, median [IQR], Celsius	34.7 [33.6-35.6]
Fluid replacement first 6 hours, median [IQR], mL	1500 [1000-2000]

© 2019 Roquet F et al. *JAMA Network Open.*

eTable 3. Adjusted Cox Model for 30-d Survival Relevant Interactions

30-day survival interactions	Complete cases (n=594)		After MI (n=897)	
SAPSII and GCS	1.003 [1.001-1.005]	0.004	1.002 [1.001-1.004]	<.001
Temperature and prothrombin time	0.99 [0.98-0.99]	0.002	0.99 [0.99-0.99]	<.001
Ratio and platelets concentrate	0.70 [0.53-0.92]	0.01		NS
Ratio and fibrinogen concentrate	1.20 [1.04-1.38]	0.01		NS
Fibrinogen concentrate and prothrombin time	1.004 [1.001-1.007]	0.001		NS

© 2019 Roquet F et al. *JAMA Network Open.*

eTable 4. Adjusted Cox Model for 6-h Survival

**6-hour survival predictors**	**Complete cases (n=594)**		**Multiple imputation (n=897)**	
	Hazard ratio [CI95%]	P Value	Hazard ratio [CI95%]	P Value
SAPSII	1.04 [1.01-1.06]	0.002	1.02 [1.01-1.04]	0.003
Intentionality				
Self-inflicted versus unintentional	0.27 [0.11-0.66]	0.004	0.64 [0.4-1.02]	0.06
Assault versus unintentional	0.24 [0.02-3]	0.27	0.37 [0.15-0.92]	0.03
Prehospital or ER cardiac arrest	4.09 [1.33-12.62]	0.01	5.44 [2.31-12.82]	<.001
Prothrombin time (PT)	1.7 [1.12-2.59]	0.01	0.99 [0.97-1]	0.08
Capillary hemoglobin - g/dL	0.83 [0.72-0.96]	0.01	0.92 [0.84-1.01]	0.09
Temperature	1.86 [1.26-2.75]	0.002	1.06 [0.93-1.21]	0.34
Lactatemia - mmol/L	1.09 [1.02-1.17]	0.01	1.08 [1.03-1.13]	0.002
Fibrinogen concentrate administration	0.93 [0.8-1.07]	0.01	0.88 [0.81-0.96]	0.004
Prehospital or emergency room vasopressor	0.41 [0.17-1.02]	0.06	0.85 [0.51-1.43]	0.29
Fluid replacement first 6 hours (crystalloids and colloids)- mL	0.99 [0.99-0.99]	<.001	1 [1-1]	0.18
Platelets concentrate	0.53 [0.31-0.9]	0.02	0.69 [0.55-0.86]	0.001
Orthopedic surgery	0.12 [0.02-0.67]	0.02	0.13 [0.03-0.52]	0.004
**High ratio versus low ratio**	**0.71 [0.35-1.43]**	**0.34**	**0.91 [0.61-1.35]**	**0.74**
Prothrombin time and temperature interaction	0.98 [0.97-1]	0.01		NS
Prothrombin time and fluid replacement interaction	1.01 [1.01-1.01]	0.007		NS

© 2019 Roquet F et al. *JAMA Network Open.*

eTable 5. Adjusted Cox Model for 24-h Survival

**24-hour survival predictors**	**Complete cases (n=594)**		**After MI (n=897)**	
	HR [CI95%]	P Value	HR [CI95%]	P Value
SAPSII	1.03 [1.01-1.04]	<0.001	1.03 [1.02-1.04]	<0.001
ISS	1.00 [0.99-1.02]	0.71	1.01 [0.99-1.01]	0.23
Intentionality				
Self-inflicted versus unintentional	0.64 [0.38-1.09]	0.12	0.79 [0.56-1.13]	0.19
Assault versus unintentional	0.34 [0.14-0.85]	0.02	0.55 [0.32-0.97]	0.04
GCS	0.97 [0.93-1.01]	0.12	1.01 [0.98-1.04]	0.63
Prehospital or ER cardiac arrest	2.67 [1.42-5.01]	0.002	3.72 [2.38-5.82]	<0.001
Prothrombin time (PT)	0.94 [0.93-0.96]	<0.001	0.97 [0.95-0.98]	<0.001
Capillary hemoglobin - g/dL	0.90 [0.82-0.99]	0.04	0.96 [0.89-1.03]	0.24
Temperature	0.94 [0.82-1.08]	0.38	0.98 [0.90-1.08]	0.72
Fibrinogen concentrate administration	0.72 [0.64-0.81]	<0.001	0.86 [0.78-0.95]	0.003
Prehospital or emergency room vasopressor	0.99 [0.55-1.79]	0.99	1.15 [0.77-1.71]	0.50
Fluid replacement first 6 hours (crystalloids and colloids)- mL	0.99 [0.99-0.99]	0.001	0.99 [0.99-1.00]	0.06
Platelets concentrate	1.17 [1.01-1.35]	0.03	1.02 [0.91-1.15]	0.71
Orthopedic surgery	0.24 [0.11-0.52]	<0.001	0.26 [0.14-0.47]	<0.001
Vascular surgery	0.49 [0.30-0.80]	0.004	0.56 [0.40-0.79]	<0.001
**High ratio versus low ratio**	**0.75 [0.48-1.18]**	**0.21**	**0.79 [0.59-1.06]**	**0.11**
Fibrinogen concentrate and prothrombin time interaction	1.01 [1.00-1.01]	<0.001	1.003 [1.000-1.006]	0.04

© 2019 Roquet F et al. *JAMA Network Open.*

eFigure 1. Receiver Operating Characteristics (ROC) Curve of the Model Selected for 30-d Survival

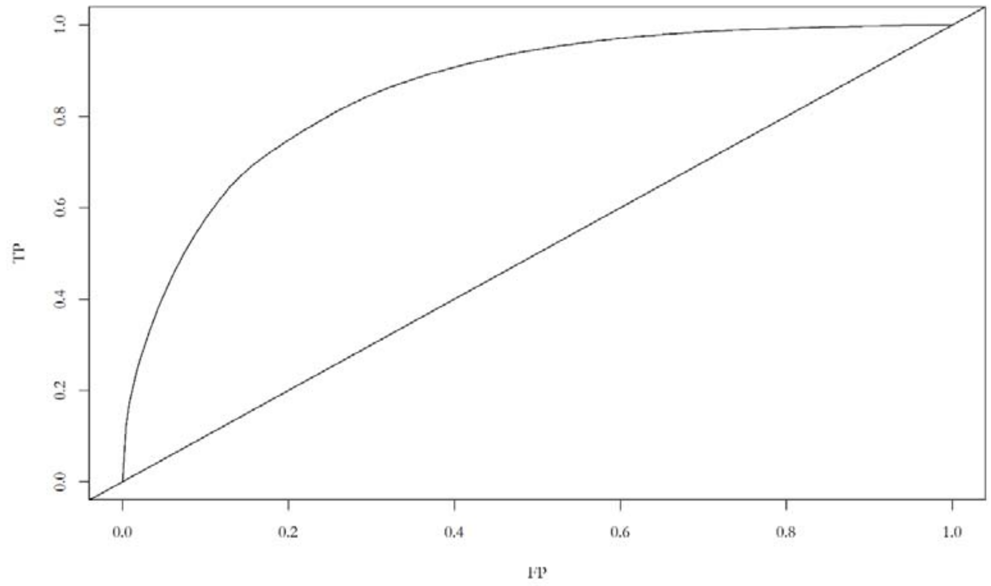

© 2019 Roquet F et al. *JAMA Network Open.*

eFigure 2. Sensitivity Analyses With Different Ratio Cutoffs (Cox Model on Complete Cases)

Low hazard ratio favors high FFP:PRBC transfusion ratio.

Variation in the transfusion ratio threshold (FFP:PRBC)

	High ratio		Low ratio		*Hazard Ratio [95% CI]*
	**Dead**	**Survival**	**Dead**	**Survival**	
1:2	218	445	83	151	0.76 [0.46, 1.25]
3:5	188	399	113	197	0.68 [0.43, 1.09]
2:3	155	351	146	245	0.57 [0.33, 0.97]
3:4	129	292	172	304	0.63 [0.40, 0.99]
4:5	122	266	179	330	0.70 [0.43, 1.14]

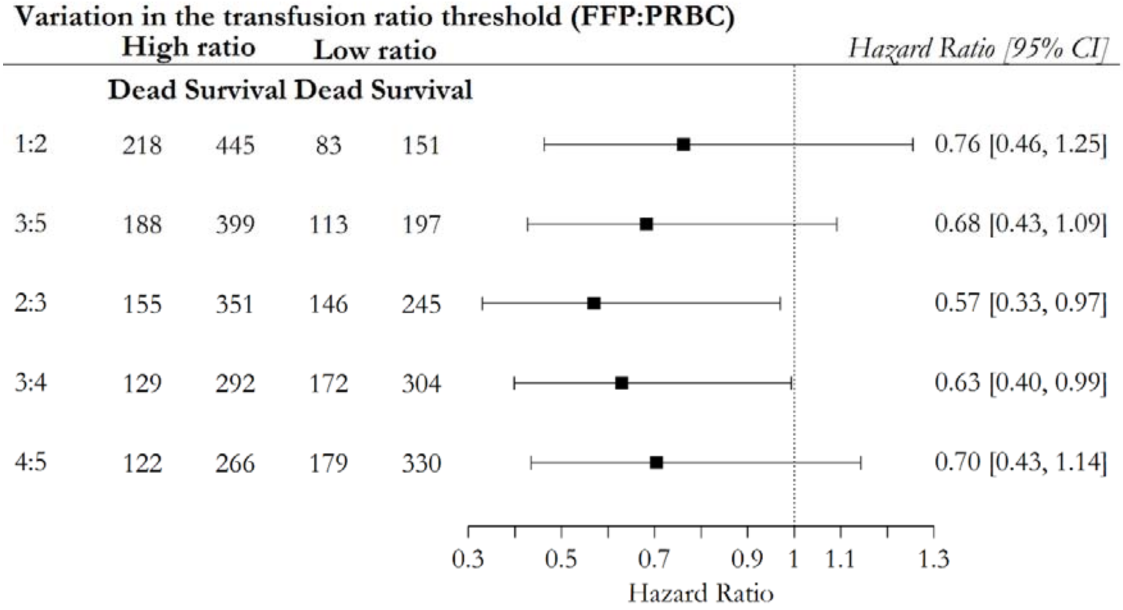

© 2019 Roquet F et al. *JAMA Network Open.*

eReferences.

1. Baker SP, O'Neill B, Haddon W, Long WB. The injury severity score: a method for describing patients with multiple injuries and evaluating emergency care. *J Trauma*. 1974;14(3):187-196.

2. Gall J-RL, Lemeshow S, Saulnier F. A New Simplified Acute Physiology Score (SAPS II) Based on a European/North American Multicenter Study. *JAMA*. 1993;270(24):2957-2963. doi:10.1001/jama.1993.03510240069035

3. Teasdale G, Jennett B. Assessment and prognosis of coma after head injury. *Acta Neurochir (Wien)*. 1976;34(1-4):45-55.

© 2019 Roquet F et al. *JAMA Network Open.*
